# Clinical Value and Prognosis of C Reactive Protein to Lymphocyte Ratio in Severe Aneurysmal Subarachnoid Hemorrhage

**DOI:** 10.3389/fneur.2022.868764

**Published:** 2022-06-13

**Authors:** Qingqing Zhang, Gaoqi Zhang, Lintao Wang, Wanwan Zhang, Fandi Hou, Zhanqiang Zheng, Yong Guo, Zhongcan Chen, Juha Hernesniemi, Hugo Andrade-Barazarte, Guang Feng, Jianjun Gu

**Affiliations:** ^1^Department of Neurosurgery, Henan University People's Hospital, Henan Provincial People's Hospital, Zhengzhou, China; ^2^School of Clinical Medicine, Henan University, Kaifeng, China; ^3^Department of Neurosurgery, Henan Provincial People's Hospital, Zhengzhou University People's Hospital, Zhengzhou, China

**Keywords:** aneurysmal subarachnoid hemorrhage (aSAH), C-reactive protein (CRP), lymphocytes, C-reactive protein to lymphocyte ratio (CLR), prognosis, outcome

## Abstract

**Objective:**

To investigate the relationship between CLR and disease severity and clinical prognosis of aSAH.

**Methods:**

The authors retrospectively analyzed the clinical data of 221 patients with aSAH, who were admitted to the intensive care unit from January 2017 to December 2020. The indicators of inflammatory factors in the first blood routine examination within 48 h of bleeding were obtained. The prognosis was evaluated by mRS score at discharge, mRS>2 was a poor outcome. Through the receiver operating characteristic (ROC) curve, the area under the curve was calculated and the predicted values of inflammatory factors (CLR, CRP, WBC, and neutrophils) were compared. Univariate and multivariable logistic regression analyses were used to evaluate the relationship between CLR and the clinical prognosis of patients. ROC curve analysis was performed to determine the optimal cut-off threshold, sensitivity, and specificity of CLR in predicting prognosis at admission.

**Results:**

According to the mRS score at discharge, 139 (62.90%) patients were classified with poor outcomes (mRS>2). The inflammatory factor with the best predictive value was CLR, which had an optimal cut-off threshold of 10.81 and an area under the ROC curve of 0.840 (95%CI.788–0.892, *P* < 0.001). Multivariable Logistic regression analysis showed that the Modified Fisher grade, Hunt-Hess grade, and CLR at admission were independent risk factors for poor outcomes of patients with aSAH (*P* < 0.05). According to Hunt-Hess grade, patients were divided into a mild group (Hunt-Hess ≤ 3) and a severe group (Hunt-Hess > 3), and the CLR value was significantly higher in severe patients with aSAH than in mild patients. The optimal cut-off threshold of CLR in the severe group was 6.87, and the area under the ROC curve was 0.838 (95% CI.752–0.925, *P* < 0.001).

**Conclusions:**

The CLR value at the admission of patients with aSAH was significantly associated with Hunt-Hess grade, The higher Hunt-Hess grade, the higher the CL R-value, and the worse the prognosis. Early CLR value can be considered as a feasible biomarker to predict the clinical prognosis of patients with aSAH.

## Introduction

Aneurysmal subarachnoid hemorrhage (aSAH) has a sudden onset and rapid progression and has high clinical mortality in clinical practice. Previous studies have shown that the mortality rate of aSAH is still as high as 66.7% (1), and the highest rate of permanent disability is about 50% (2). Although mortality and permanent disability rates have decreased with the rapid development of neuro-intensive care techniques, mortality and disability rates remain high for patients with severe conditions at admission and higher Hunt-Hess grades.

There are many factors affecting the clinical prognosis of aSAH patients. In recent years, many clinical studies have used white blood cells, neutrophils, lymphocytes, and C-reactive protein to predict the relationship between them and the clinical prognosis of patients with aSAH (3–7). Güresir et al. (3) reported that the initial inflammatory response was an independent predictor of the clinical prognosis of patients with aSAH. Al-Mufti et al. (7) reported that white blood cell (WBC) count at admission could predict delayed cerebral ischemia after aneurysmal subarachnoid hemorrhage. The higher the WBC count, the greater the probability of delayed cerebral ischemia (DCI), resulting in poor outcomes. However, most of these clinical predictors have low specificity and sensitivity. Therefore, inflammatory factors that can accurately predict the clinical prognosis of patients with aSAH at an early stage are still of great significance for clinical treatment.

C-reactive protein (CRP) is an acute phase protein (8, 9), which is produced by the liver and stimulated by various cells under the action of the body under trauma or inflammatory factors (10, 11). Lymphocytes play a role in promoting the growth and regulation of endothelial cells, which will be consumed in large quantities when the body is traumatized (12). The ratio of C-reactive protein to lymphocytes (CLR) plays a key role in the prognosis of tumors, pancreatic cancer, colorectal cancer, and other related diseases, but there is no study on patients with aSAH. This study mainly explores the clinical value of CLR in patients with aSAH and its relationship with clinical prognosis.

## Materials and Methods

### Patient Population

A retrospective analysis of 307 patients with aSAH was admitted to the intensive care unit from January 2017 to December 2020 was performed. There were 221 patients, who met the criteria, aged 29–88,135 (61.09%) females and 86 (38.91%) males, and with an average age of 58.87 ± 11.99. The inclusion criteria for enrollment were as follows: (1) Acute onset, arrived at our hospital within 48 h and underwent laboratory examination, (2) diagnosed with spontaneous SAH by computed tomography (CT), and the diagnosis of the aneurysm was achieved by computed tomography angiography (CTA) or digital subtraction angiography (DSA), and (3) all of them underwent interventional or surgical clipping treatment.

The study exclusion criteria were as follows: (1) no aneurysm was found by CTA or DSA, (2) more than 48 h after reaching our hospital, (3) patients with incomplete clinical data, (4) complicated with infection, immune dysfunction, blood system diseases, organ function damage, experienced surgery, or major infectious diseases in the past 1 month. This study was approved by the Institutional Review Board. We did not sign the written informed consent, but we obtained the consent of the patient or his family through a telephone interview.

### Data Collection

The basic clinical data of all patients were collected, including gender, age, hypertension, diabetes, smoking, drinking history, history of cerebral infarction, mRS at admission, mRS at discharge, modified Fisher score, and Hunt-Hess grade. Venous blood samples were collected within 48 h at admission and stored in tubes containing various anticoagulants for routine blood tests. Routine blood examinations, including examinations of the white blood cell count (reference range, 3.5–9.5 10^9^ /L), neutrophil count (reference range, 1.8–6.3 10^9^ /L), lymphocyte count (reference range, 1.1–3.2 10^9^ /L), and C-reactive protein count (reference range, 0–10 mg/L), were performed for all patients by the routine laboratory assays. The CLR is defined as the number of C-reactive proteins divided by the number of lymphocytes. The status of patients at admission was evaluated according to the modified Fisher scale and Hunt-Hess classification. All subjects were treated with surgery or interventional therapy and discharged from the hospital with a modified Rankin Scale (mRS) score of < 2 for good outcomes and > 3 for poor outcomes.

### Statistical Analysis

The SPSS 21.0 statistical software was used for data analysis. Kolmogorov-Smirnov method was used to test whether the data were by the normal distribution. Mean ± standard deviation ($\bar{x}\pm$s) was used for measurement by a normal distribution, and a *t*-test was used for comparison between groups. Median (M) and quartile (P25, P75) were used for measurement data that did not conform to the normal distribution, and the Wilcoxon rank test was used for comparison between groups. Several cases and constituent ratios were used for enumeration data, χ2 test was used for comparison between groups. Factors *P* < 0.05 in univariate analysis results were included as dependent variables in multivariable Logistic regression analysis, and receiver operating characteristic (ROC) curve analysis was performed to determine the optimal cut-off value, sensitivity, and specificity of CLR for predicting prognosis at admission. *P* < 0.05 was considered statistically significant (13).

## Results

**Figure 1:**
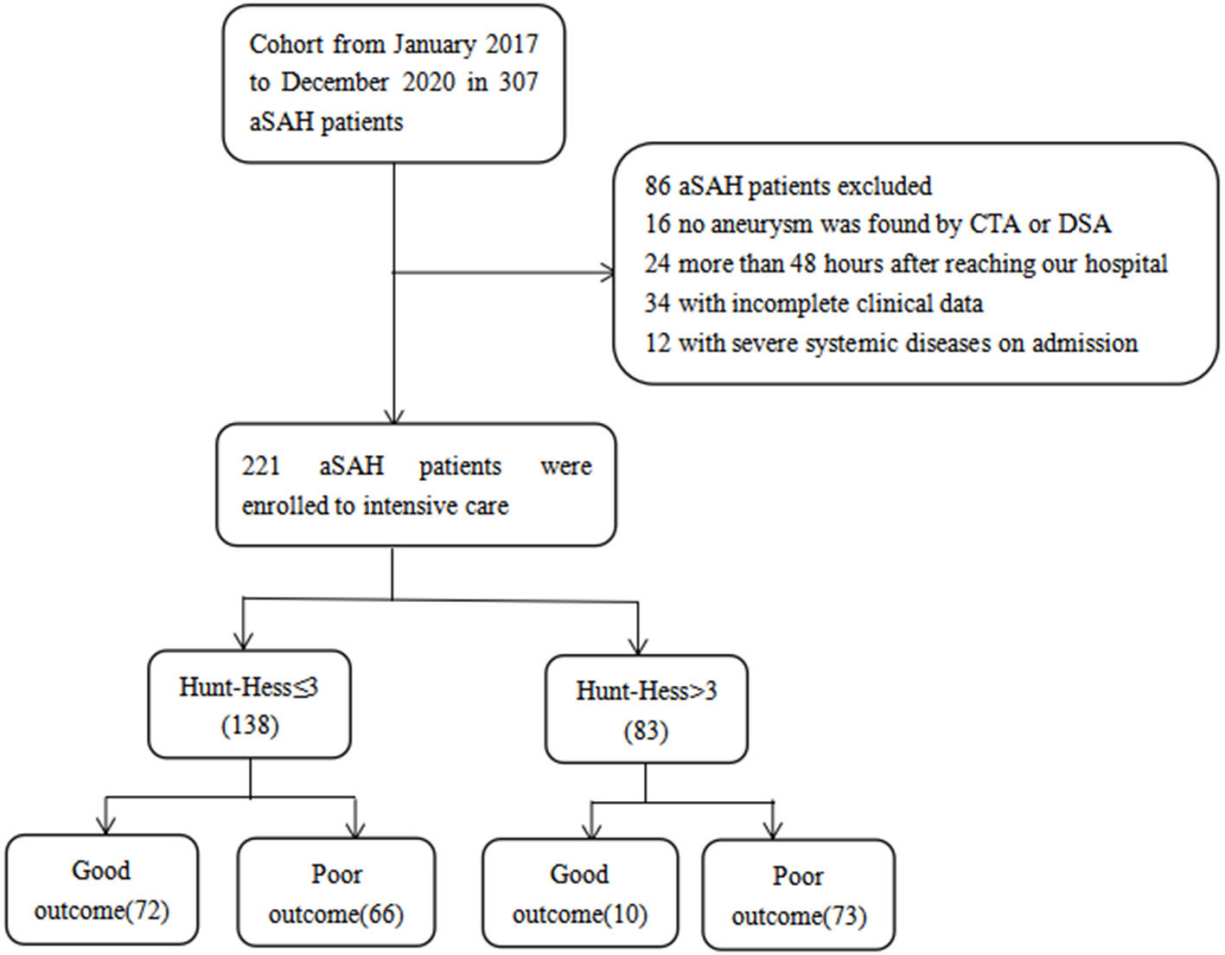
Flowchart illustrating the patient cohort included in this study.

A total of 221 patients with aSAH admitted to the intensive care unit were enrolled, 82 patients with good outcomes and 139 patients with poor outcomes, including 14 deaths. According to Hunt-Hess's grade at admission, 138 cases were mild (Hunt-Hess ≤ 3), 72 cases had good outcomes, and 66 cases had poor outcomes. There were 83 cases noted to be severe (Hunt-Hess > 3), with good outcomes in 10 cases and poor outcomes in 73 cases (Figure 1).

This study included 82 patients with aSAH in the good outcome group and 139 patients with aSAH in the poor outcome group, including 54 females and 28 males aged 55.72 ± 11.30 years in the good outcome group and 81 females and 58 males aged 60.29 ± 12.59 years in the poor outcome group. There were no significant differences in gender, smoking, drinking, or location of aneurysms between good and poor outcome groups (*P* > 0.05). There were statistically significant differences in age, diabetes or hypertension, cerebral infarction, Fisher score at admission, and Hunt-Hess grade between good and poor outcome groups (*P* < 0.05). However, the difference in inflammatory indexes (leukocytes, neutrophils, lymphocytes, and CRP) between the two groups was also statistically significant (*P* < 0.05). In addition, the CLR value of the poor outcome group (35.92 ± 41.67) was higher than that of patients in the good outcome group (4.44 ± 7.74), and the difference was statistically significant (*P* < 0.05), as shown in Table 1.

**Table 1:** Univariate analysis table affecting the clinical prognosis of patients with Aneurysmal subarachnoid hemorrhage (aSAH).

**Variables**	**Total patients (221)**		***P* value**
	**Good outcome (82)**	**Poor outcome (139)**	
Gender			0.264
Female	54 (65.85%)	81 (58.27%)	
Male	28 (34.15%)	58 (41.73%)	
Age, years	55.72 ± 11.30	60.29 ± 12.59	0.006
Smoking	17 (20.73%)	30 (21.58%)	0.881
Drinking	15 (18.29%)	29 (20.86%)	0.644
Diabetes	0 (0.00%)	12 (8.63%)	0.015
Hypertension	38 (46.34%)	90 (64.75%)	0.007
Cerebral infarction	4 (4.88%)	21 (53.85%)	0.034
Coronary artery disease	4 (4.88%)	19 (13.67%)	0.066
Modified fisher grade			<0.001
Grade 0,1,2	74 (90.24%)	66 (47.48%)	
Grade 3,4	8 (9.76%)	73 (52.52%)	
Hunt-Hess grade			<0.001
Grade 1,2,3	72 (87.80%)	66 (47.48%)	
Grade 4,5	10 (12.20%)	73 (52.52%)	
Location			0.741
Anterior circulation	42 (51.22%)	68 (48.92%)	
Posterior circulation	40 (48.78%)	71 (51.08%)	
Inflammation index			
WBC	11.28 ± 3.87	13.24 ± 4.39	0.001
Neutrophil	9.69 ± 3.73	11.57 ± 4.44	0.002
Lymphocyte	1.11 ± 0.53	1.00 ± 0.56	0.026
CRP	4.21 ± 4.84	28.47 ± 32.76	<0.001
CLR	4.44 ± 7.74	35.92 ± 41.67	<0.001

*WBC, white blood cell; CRP, C-reactive protein; CLR, C-reactive protein to lymphocyte ratio*.

Factors associated with poor outcomes in Table 1 were included in multivariable logistic regression analysis after excluding some confounding factors [due to the high collinearity between CRP and CLR (VIF > 10 after log-conversion), CRP was excluded]. It was found that the improved Fisher score, Hunt-Hess grade, and CLR at admission were independent risk factors for poor outcomes of patients with aSAH (*P* < 0.05), as shown in Table 2.

**Table 2:** Multivariable logistic regression analysis of poor clinical prognosis of aSAH.

**Variables**	**Total patients (221)**		
	**OR**	**95% CI**	***P* value**
Age, years	0.988	0.949–1.028	0.551
Cerebral infarction	3.157	0.777–12.831	0.108
Hypertension	1.228	0.516–2.918	0.643
Modified fisher grade	6.023	2.045–17.735	0.001
Hunt-Hess grade	5.071	1.840–13.978	0.002
WBC	1.028	0.400–2.646	0.954
Neutrophil	0.958	0.373–2.462	0.929
Lymphocyte	1.495	0.411–5.441	0.542
CLR	1.159	1.087–1.236	<0.001

*WBC, white blood cel; CLR, C-reactive protein to lymphocyte ratio*.

**Figure 2:**
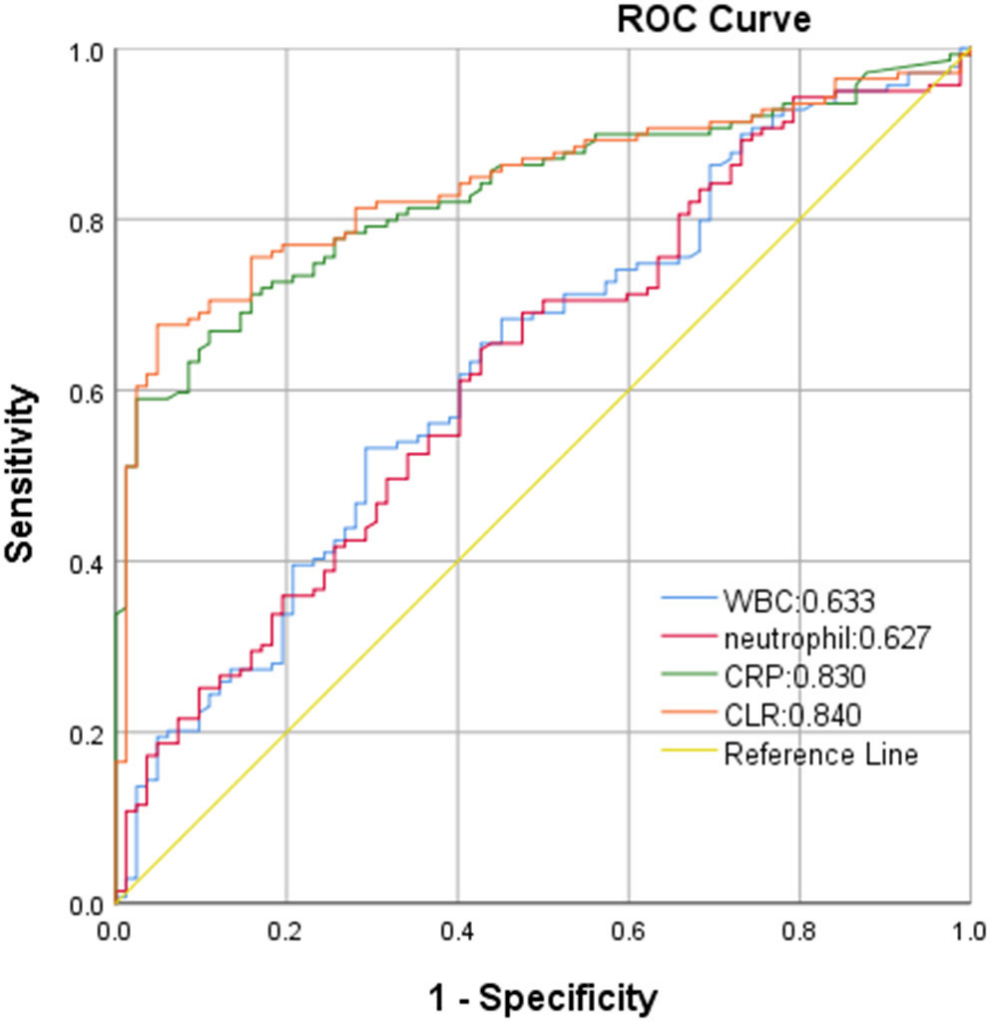
Receiver operating characteristics (ROC) curve of CLR in predicting poor outcome of aSAH patients.

ROC curve analysis showed that white blood cells, neutrophils, CRP, and CLR were 0.633, 0.627, 0.830, and 0.840, respectively. CLR had the highest accuracy in predicting the clinical prognosis of patients with aSAH. The optimal cut-off threshold of CLR was 10.81, the area under the ROC curve was 0.840 (95 % CI 0.788–0.892, *P* < 0.001), the maximum Youden index was 0.627, the sensitivity was 0.676, and the specificity was 0.951 (Figure 2).

By comparing the mild with the severe group, it was found that there was a significant correlation between disease severity and patients' age, hypertension, coronary heart disease, history of cerebral infarction, modified Fisher score, WBC, neutrophils, CRP, and CLR (*P* < 0.05). The CLR value (33.96 ± 44.85) was significantly higher in the severe group than in the mild group (18.39 ± 29.36) (Table 3). Univariate analysis of the severe group showed that the age of patients with poor outcomes was generally higher than that of patients with good outcomes. The CLR value of patients with poor outcomes (38.20 ± 46.24) was higher than that of patients with good outcomes (2.94 ± 2.05), and the difference had statistical significance (*P* < 0.05, Table 4).

**Table 3:** Univariate analysis of clinical characteristics of patients in mild and severe groups.

**Variables**	**Hunt-Hess≤3 (138)**	**Hunt-Hess>3 (83)**	***P* value**
Gender			0.513
Female	82 (59.42%)	53 (63.86%)	
Male	56 (40.58%)	30 (36.14%)	
Age, years	56.63 ± 11.94	62.59 ± 11.19	<0.001
Smoking	3,021.74%	17 (20.48%)	0.825
Drinking	25 (18.12%)	19 (22.89%)	0.389
Diabetes	5 (3.62%)	7 (8.43%)	0.222
Hypertension	63 (45.65%)	65 (78.31%)	<0.001
Cerebral infarction	8 (5.80%)	17 (20.48%)	0.001
Coronary artery disease	8 (5.80%)	15 (18.07%)	0.004
Modified fisher grade			<0.001
Grade 0,1,2	108 (78.26%)	32 (38.55)	
Grade 3,4	30 (21.74%)	51 (61.45%)	
Location			0.306
Anterior circulation	65 (47.10%)	45 (54.22%)	
Posterior circulation	73 (52.90%)	38 (45.78%)	
Inflammation index			
WBC	11.77 ± 4.10	13.75 ± 4.36	0.001
Neutrophil	10.21 ± 4.02	11.98 ± 4.49	0.004
Lymphocyte	1.05 ± 0.52	1.04 ± 0.60	0.542
CRP	13.84 ± 18.03	28.82 ± 38.92	0.004
CLR	18.39 ± 29.36	33.96 ± 44.85	0.005

*WBC, white blood cell; CRP, C-reactive protein; CLR, C-reactive protein to lymphocyte ratio*.

**Table 4:** Clinical characteristics of clinical prognosis in patients with aSAH with Hunt-Hess of >3.

**Variables**	**Hunt-Hess>3 (83)**		***P* value**
	**Good outcome (10)**	**Poor outcome (73)**	
Gender			0.138
Female	9 (90.00%)	44 (60.27%)	
Male	1 (10.00%)	29 (39.73%)	
Age, years	62.00 ± 8.731	62.67 ± 11.528	0.014
Smoking	0 (0.00%)	17 (23.29%)	0.196
Drinking	1 (10.00%)	18 (24.66%)	0.526
Diabetes	0 (0.00%)	7 (9.59%)	0.677
Hypertension	8 (80.00%)	57 (78.08%)	1.000
Cerebral infarction	2 (20.00%)	15 (20.55%)	1.000
Coronary artery disease	1 (10.00%)	14 (19.18%)	0.788
Modified fisher grade			0.255
Grade 0,1,2	6 (60.00%)	26 (35.62%)	
Grade 3,4	4 (40.00%)	47 (64.38%)	
Location			
Anterior circulation	6 (60.00%)	39 (53.42%)	0.958
Posterior circulation	4 (40.00%)	34 (46.58%)	
Inflammation index			
WBC	10.83 ± 3.29	14.15 ± 4.35	0.028
Neutrophil	9.46 ± 3.53	12.32 ± 4.51	0.050
Lymphocyte	1.08 ± 0.45	1.03 ± 0.62	0.543
CRP	3.33 ± 3.18	32.31 ± 40.26	0.001
CLR	2.94 ± 2.05	38.20 ± 46.24	0.001

*WBC, white blood cell; CRP, C-reactive protein; CLR, C-reactive protein to lymphocyte ratio*.

The factors associated with poor outcomes in the severe group in Table 4 are included in the multivariable logistic regression analysis after excluding some confounding factors [due to the high collinearity between CRP and CLR (VIF > 10 after log-conversion), CRP was excluded]. Finally, CLR was found to be an independent risk factor for poor outcomes in patients with severe aSAH (Table 5).

**Table 5:** Multivariable logistic regression analysis of poor clinical prognosis in severe group (Hunt-Hess > 3).

**Variables**	**severe group (83)**		***P* value**
	**OR**	**95% CI**	
Age, years	1.053	0.973–1.139	0.201
WBC	2.850	0.589–13.784	0.193
Neutrophil	0.414	0.089–1920	0.260
CLR	1.280	1.002–1.635	0.048

*WBC, white blood cell; CLR, C-reactive protein to lymphocyte ratio*.

**Figure 3:**
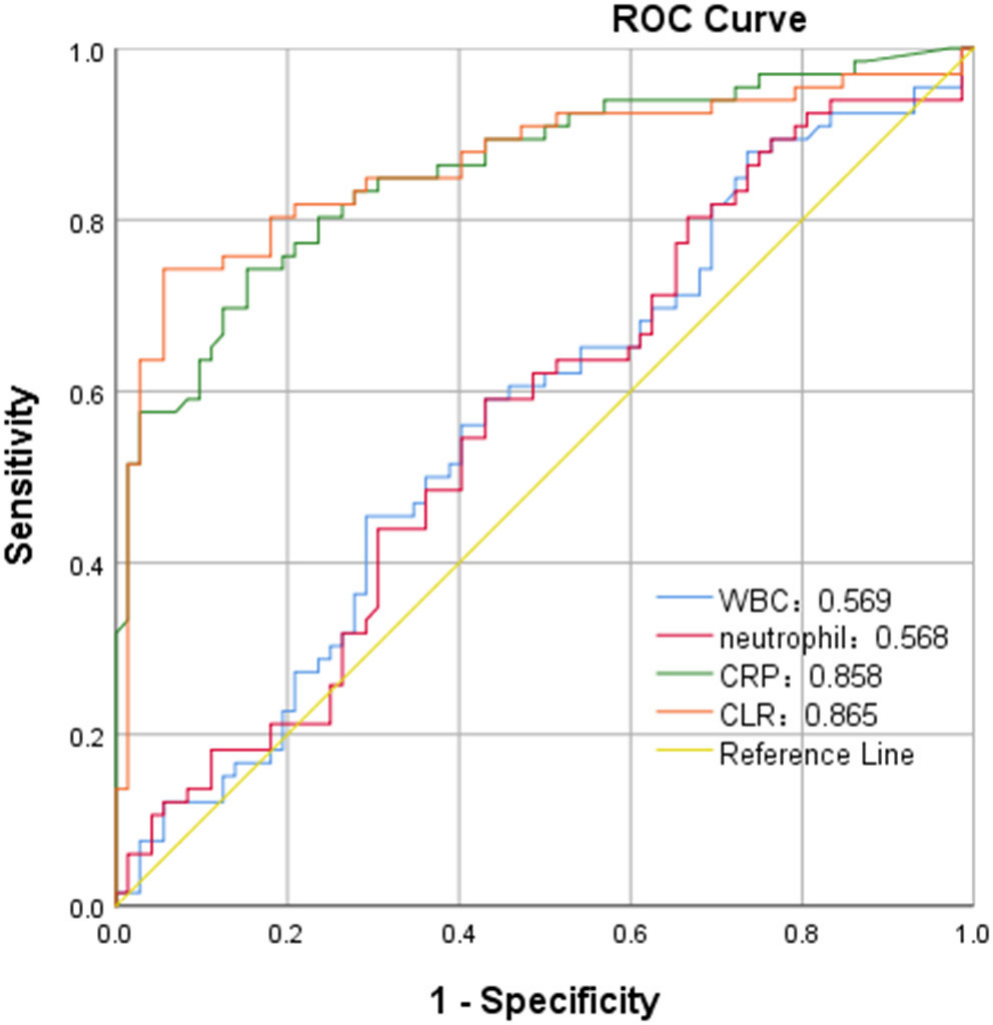
ROC curve of C-reactive protein to lymphocyte ratio (CLR) in predicting poor outcome of patients with aSAH in mild group (Hunt-Hess of ≤ 3).

**Figure 4:**
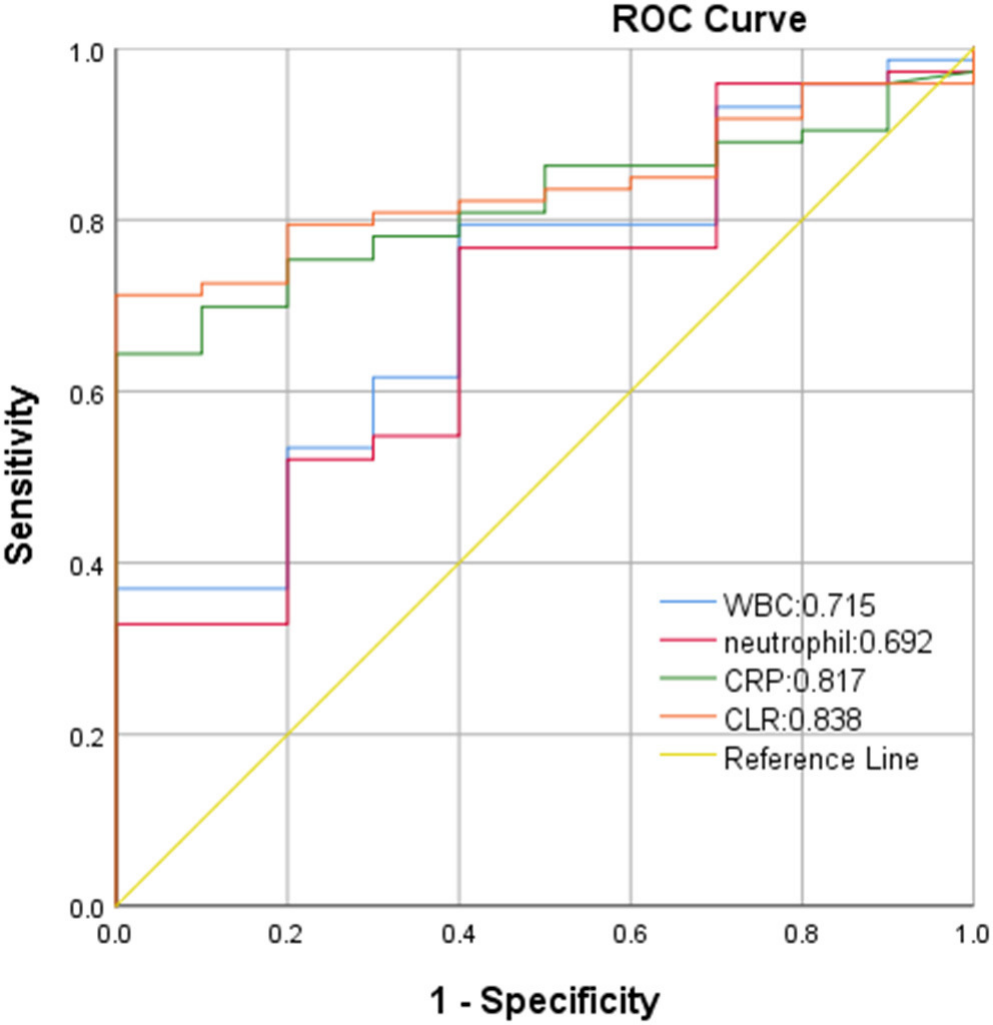
ROC curve of CLR in predicting poor outcome of patients with aSAH in severe group (Hunt-Hess of > 3).

In ROC curve analysis, the white blood cells, neutrophils, CRP, and CLR values of patients in the mild group (Hunt-Hess ≤ 3) were 0.569, 0.568, 0.858, and 0.865, respectively. The optimal cut-off threshold of CLR was 10.81, the area under the ROC curve was 0.865 (95% CL0.799–0.931, *P* < 0.001), and the maximum Youden index was 0.686, and its sensitivity was 0.742 and specificity was 0.944 (Figure 3). The white blood cell, neutrophil, CRP, and CLR values of patients in the severe group (Hunt-Hess >3) were 0.715, 0.692, 0.817, and 0.838, respectively. The optimal cut-off threshold of CLR was 6.87, the area under the ROC curve was 0.838 (95% CL0.752–0.925, *P* < 0.001), and the maximum Youden index was 0.712, its sensitivity was 0.712 and the specificity was 1 (Figure 4).

## Discussion

From 221 patients admitted to intensive care due to rapid and severe disease progression at admission, we found that among 83 patients with a Hunt-Hess score of >3 that an elevated baseline systemic inflammatory response was an independent risk factor for poor outcome. Although some patients have a better neurological grade on admission, there is still an unpredictable risk of deterioration during treatment, leading to a poor outcome (14, 15). Therefore, the ability to accurately predict prognosis, especially for patients with severe disease and higher Hunt-Hess grade at admission, is important to adjust the management and treatment of patients promptly. Finally, with stepwise validation at baseline, we found that CLR was the strongest independent predictor of prognosis in patients with aSAH. It is a simple and approachable method for early predictability of poor outcomes in patients with aSAH.

The aSAH is a sudden and rapidly progressive disease, especially in patients with severe disease at admission and long hospital stay, it is still a clinical challenge to predict the prognosis of aSAH. After an aneurysm rupture, blood deposition into the subarachnoid cavity will stimulate the brain tissue and activate the immune regulatory cells in the central nervous system, and a large number of inflammatory cells will enter the subarachnoid cavity, which will rapidly cause an inflammatory cascade reaction (16, 17). At present, in clinical practice, inflammatory factors at admission are some of the data we can utilize, and these inflammatory factors are all part of the identifiable data at the admission of patients, avoiding the influencing factors known as sequelae left in the clinical course after aSAH.

Gaastra et al. (18) reported that CRP is an independent predictor of postoperative functional outcome in patients with aSAH, and elevated CRP values at the initial stage of bleeding are closely related to the functional outcome of clinical prognosis (19, 20). CRP is an acute-phase protein produced by the liver (8, 9). Under the action of trauma or inflammatory factors, the liver will rapidly produce CRP and release it into the peripheral blood. At this time, CRP in peripheral blood is elevated (12, 16–18). However, CRP is a non-specific inflammatory marker that can be elevated in the presence of any tissue damage (10, 11), therefore, its clinical value can be improved in combination with other inflammatory indicators.

On the other hand, when the central nervous system is stimulated, the immune system will be activated and a large number of lymphocytes will be released, which can reduce the damage to brain tissue through antigen recognition, cell activation, and immune killing (16). Frösen et al. (21) compared 42 cases of ruptured and 24 cases of unruptured aneurysms in histology and found that lymphocytes actively participate in the inflammatory cascade reaction of the vascular wall of the aneurysm. When aneurysm ruptures, excessive depletion of lymphocytes, results in a decrease in the number of lymphocytes, which is also considered to be a sign of aggravating brain injury with poor clinical outcomes (16). While CLR is a new inflammatory ratio and has been used as one of the prognostic markers of pancreatic and rectal cancer surgery, tumor, and strangulated abdominal hernia (intestinal ischemia) in current studies (22–25). Fan et al. (23) compared the combination of six inflammatory markers, namely neutrophil/lymphocyte ratio (NLR), platelet/lymphocyte ratio (PLR), C-reactive protein/albumin ratio (CAR), neutrophil/albumin ratio (NAR), platelet/albumin ratio (PAR), and C-reactive protein/lymphocyte ratio (CLR) to predict the accuracy of poor survival rate of pancreatic cancer, and found that CLR was more accurate than NLR, PLR, CAR, NAR and PAR in predicting the survival rate of pancreatic cancer. While CLR has not been reported in the literature in related aspects, such as cerebral hemorrhage and traumatic brain injury. In this study, increased CLR value was related to increased risk for poor outcomes of patients with aSAH, and higher accuracy of CLR relative to WBC, neutrophils, and lymphocytes was observed to predict the prognosis of patients with aSAH.

Our study also found that CLR value is closely related to Hunt-Hess grade, and for patients with higher Hunt-Hess grade and more severe disease, the higher the CLR value, the worse the prognosis. Therefore, CLR values reflect early C-reactive protein excess and lymphocyte depletion in aSAH patients, which may predict preoperative severity and postoperative prognosis. Hunt-Hess grade is used to classify the clinical status of patients with aSAH to select the timing of surgery and determine the prognosis. Related literature has reported that the higher the Hunt-Hess grade of patients at admission, the worse the prognosis (26–28). Frontera et al. showed that in the severe group (Hunt-Hess > 3), patients with aSAH were more prone to ischemic brain injury in the early stage, with higher mortality and disability rates, confirming our findings (26).

However, our study had some limitations. Firstly, the number of patients enrolled in the study is small; second, our analysis is retrospective and carried out in a single center; thirdly, we did not count the blood volume of subarachnoid hemorrhage, therefore, whether the amount of lymphocyte depletion is related to the amount of blood in subarachnoid hemorrhage still requires our further study; and finally, we only investigated the prognosis of patients at discharge. Therefore, long-term perspective and multi-center trials are needed to support our results in the future.

## Conclusions

This study indicated that patients with aSAH with high levels of CLR at admission were at high risk for worse outcomes at discharge and the higher CLR value was related to the worse prognosis in patients with aSAH with the higher Hunt-Hess grade (>3), which suggests that CLR may be as a potential clinical biomarker to predict prognosis among patients with aSAH with the higher Hunt-Hess grade (>3).

## Author Contributions

JG contributed to conception and design of the study. QZ and GZ wrote the first draft of the manuscript. LW, WZ, FH, ZZ, and YG wrote sections of the manuscript. All authors contributed to manuscript revision, read, and approved the submitted version.

## Funding

This research was supported by Henan Medical Science and Technology Public Relations Program (Joint Construction) Project, Number: LHGJ20190592; by the Senior Specialist Foreign Expert Project, Number: G20190126006; and by Department of Science and Technology of Henan Province.

## Conflict of Interest

The authors declare that the research was conducted in the absence of any commercial or financial relationships that could be construed as a potential conflict of interest.

## Publisher's Note

All claims expressed in this article are solely those of the authors and do not necessarily represent those of their affiliated organizations, or those of the publisher, the editors and the reviewers. Any product that may be evaluated in this article, or claim that may be made by its manufacturer, is not guaranteed or endorsed by the publisher.
