# Corrigendum: Clinical value and prognosis of C reactive protein to lymphocyte ratio in severe aneurysmal subarachnoid hemorrhage

**DOI:** 10.3389/fneur.2022.1055700

**Published:** 2022-12-01

**Authors:** Qingqing Zhang, Gaoqi Zhang, Lintao Wang, Wanwan Zhang, Fandi Hou, Zhanqiang Zheng, Yong Guo, Zhongcan Chen, Juha Hernesniemi, Hugo Andrade-Barazarte, Guang Feng, Jianjun Gu

**Affiliations:** ^1^Department of Neurosurgery, Henan University People's Hospital, Henan Provincial People's Hospital, Zhengzhou, China; ^2^School of Clinical Medicine, Henan University, Kaifeng, China; ^3^Department of Neurosurgery, Henan Provincial People's Hospital, Zhengzhou University People's Hospital, Zhengzhou, China

**Keywords:** aneurysmal subarachnoid hemorrhage (aSAH), C-reactive protein (CRP), lymphocytes, C-reactive protein to lymphocyte ratio (CLR), prognosis, outcome

In the published article, there was an error in the article type. Instead of “Review,” it should be “Original Research”.

The authors apologize for this error and state that this does not change the scientific conclusions of the article in any way. The original article has been updated.

## Publisher's note

All claims expressed in this article are solely those of the authors and do not necessarily represent those of their affiliated organizations, or those of the publisher, the editors and the reviewers. Any product that may be evaluated in this article, or claim that may be made by its manufacturer, is not guaranteed or endorsed by the publisher.

